# The Influence of WB-EMS-Training on the Performance of Ice Hockey Players of Different Competitive Status

**DOI:** 10.3389/fphys.2019.01136

**Published:** 2019-09-10

**Authors:** Elisabeth Schuhbeck, Christof Birkenmaier, Heike Schulte-Göcking, Andreas Pronnet, Volkmar Jansson, Bernd Wegener

**Affiliations:** ^1^Department of Orthopedic Surgery, Physical Medicine and Rehabilitation, Ludwig Maximilian University of Munich, Munich, Germany; ^2^Aktiva Medici Rehabilitation Center, Prien am Chiemsee, Germany

**Keywords:** WB-EMS, slap shot, shot speed, counter-movement-jump, isokinetic strength, knee extensor, subjective performance, ice hockey

## Abstract

**Purpose:**

The aim of this study was to examine the influence of long-term whole-body electromyostimulation (WB-EMS) training in addition to standard ice hockey training in the following areas: shot speed, counter-movement-jump (CMJ) height and power, 10 m-sprint, isokinetic maximum force at 60 and 300°/s of the knee extensor muscle and subjective performance. The purpose was further to check, whether competitive status influenced the extent of response to WB-EMS and whether WB-EMS would hypothetically be a suitable method to reduce injury rate.

**Methods:**

Thirty male amateur ice hockey players participated in this study. They were divided into two cross-over groups (Group A and Group B). EMS sessions were carried out once a week for 12 weeks for each group with a subsequent 4 week EMS pause. The sessions consisted of 20 min electromyostimulation with 150 contractions (4 s duration, 85 Hz). Shot speed of slap shot was measured with Sportradar 1503. Jumping ability was determined with a ground reaction force platform (GRFP). Sprint time for 10 m skate was recorded using an infrared photo sensor. Isokinetic force of the knee extensor muscle was detected with Isomed 2000 at two different angular velocities (60 and 300°/s) and the subjective performance was collected using a questionnaire.

**Results:**

After 12 weeks of WB-EMS training jumping power increased significantly for the WB-EMS groups by 5.15%, 10 m skating time decreased significantly by 5%, and maximum isokinetic force at 300°/s increased significantly by 7% (all *p* < 0.05). In contrast post training shot speed showed no significant change. Isokinetic torque at 60°/s and vertical jump height were collected as secondary variables and showed increases of 5.45 and 15.15%, respectively. After finishing the WB-EMS and continuing the normal training, it was shown that the training effect regressed.

**Conclusion:**

This study demonstrated that WB-EMS training significantly decreased 10 m skating time and increased jumping power and maximum isokinetic force at 300°/s. We conclude that with additional WB-EMS training, an increase in performance might also be achieved for athletes in lower leagues. Due to the higher training potential of leisure athletes, the effect is probably even more pronounced than would be expected for competitive athletes.

## Introduction

In medicine electromyostimulation has long been an established method for supplementing rehabilitation concepts. It is utilized to increase the functional efficiency of patients with gonarthrosis (Banzer, 2016; Klimkiewicz et al., 2016), to reduce back pain (Kemmler et al., 2017) and to serve as a well-documented procedure for rehabilitation of paresis in stroke patients (Bock, 2018). Furthermore is was shown that whole-body electromyostimulation (WB-EMS) improves body composition in patients with hematological malignancies (Schink et al., 2018) and increases muscle mass in patients at risk for sarcopenic obesity (Kemmler et al., 2016).

During the last years, WB-EMS has found its way into strength training. Several studies on professional athletes of various kinds of sport have shown that complimentary training with WB-EMS can achieve efficient results in strength gains. Those ranged up to 19.5% in terms of jumping ability and up to 35.2% in terms of maximum force respectively, as seen in volleyball (Malatesta et al., 2003), basketball (Maffiuletti et al., 2000) and soccer players (Billot et al., 2010; Filipovic et al., 2016), swimmers (Pichon et al., 1995) as well as rugby (Babault et al., 2007) and tennis players (Maffiuletti et al., 2009).

In Filipovic et al.’s (2012) systematic review regarding EMS training on trained and untrained athletes maximum force, high-speed force, vertical jump height and sprint time were examined. The training period amounted to 3–6 weeks. Results showed an increase of maximum isokinetic strength in eccentric (37.1%) and concentric condition (41.3%) and counter-movement-jump (CMJ) performance (19.2%), such as a decrease of sprint time (4.8%). The effect on untrained athletes however, was not discussed in detail. Brocherie et al. (2005) published an EMS study on French division II ice hockey players which revealed a 41% increase of maximum power at an angular velocity of 60°/s and 49% at 300°/s and a 4.8% decrease of sprinting time and 6% decrease of jumping height. However, many of those studies show diverging not comparable results due to a smaller number of cases and shorter lengths of training periods, aberrant stimulation modes, testing procedures, and individual conditions of the subjects. Moreover nearly all of these studies discuss the effect of EMS on professional athletes.

To the best of our knowledge, no WB-EMS study has previously been published on leisure ice hockey players. We were interested in the question whether this fairly new training concept may also benefit athletes of amateur leagues. This constitutes an interesting group of athletes, as especially the pool of district league players represents a possible way to recruit young professional athletes. Since most studies have only stimulated individual muscle groups, we wanted to investigate the effect of WB-EMS training on amateur ice hockey players and investigate whether athletes’ competitive status as an indicator of their fitness level influenced the magnitude of their response to WB-EMS. Due to the greater training potential of hobby athletes we expect them to gain higher effects on the performance increase through additional WB-EMS training.

Lastly training with WB-EMS and strength training show similar effects (Simpson and Willoughby, 1988; Colson et al., 2009). Furthermore there is a correlation between range of maximum power, body control and injury rate. In addition to equipment and fair play, specific strength training and proprioceptive training are methods to prevent injury to hip and core muscles (Weisskopf, 2010). A minimum of 6 weeks specific strength program and proprioceptive training decreases the injury rate of adductors and inguinal structures (Tyler et al., 2002). Therefore, a reduction of the injury rate could be expected from an improvement of strength and proprioception.

## Materials and Methods

### Subjects

The present study included 30 male ice hockey players aged 18–48 years (on average 27.5 ± 7.9 years) from two amateur ice hockey leagues (70% district league players and 30% hobby league players). Anthropometric data were collected (height: 1.81 ± 0.07 m; weight: 80.2 ± 12.5 kg, circumference of thigh: 58.5 ± 6 cm; upper arm: 35.6 ± 3 cm, and waistline: 88.9 ± 9 cm). The players’ average begin with ice hockey was at child’s age, their training frequency ranged from 2 trainings and 1 game per week for hobby league players to 3 trainings plus 1–2 games per week for district league players.

Before training, differences were observed between Group A and Group B. Group A athletes weighed an average of 12.98 kg more, their circumferences of thigh and upper arm were 4 and 2 cm bigger, as well as their average waistline (5 cm). Group A athletes showed higher baseline values in jump power (0.29 kW), shot speed (11.55 km/h), maximum force at 300°/s (13.11 Nm), and maximum force at 60°/s (9.12 Nm), whereas Group B athletes reached higher baseline values in jump height (3.56 cm) and lower values in sprint time (0.06 s). The athletes were assigned randomly and in equal parts to the two groups, also in terms of their league affiliation. They participated voluntarily and signed an informed consent prior to beginning with the training. The study was approved by the Ethics Committee of the Ludwig Maximilian University of Munich. It was registered with the German clinical trials register (ID: DRKS00012249) and is therefore listed in the International Clinical Trials Registry of the World Health Organization. Over the period of our study, ice hockey training continued in the same manner for all participants as usual. The players performed their hobbies the same way as before and equally in both study periods. Four athletes had to resign from the study because of injuries sustained during ice hockey training, but unrelated to WB-EMS training.

### WB-EMS-Training

In a randomized cross-over design, one group was first trained with WB-EMS in addition to normal training for 12 weeks, followed by a phase without WB-EMS for 4 weeks. During the first 6 weeks, WB-EMS training was performed in static mode, followed by a further 6 weeks of dynamic training. The second group performed WB-EMS in exactly the same fashion. The WB-EMS period consisted of a total of 12 sessions in 12 weeks, one session per week, with a duration of 20 min each. The full number of sessions was completed by every participant in the predetermined time. Training times were set in adequate time distance from ice hockey training and matches to create a sufficient recovery period. Ordinarily, the training was held separately from ice hockey training, and was set at always the same days of the week. The training plan targeted every big muscle group. Training time was divided into different sections in which specific exercises for the respective disciplines were carried out. As overview:

*Sprint*: Abductor training, side steps, static, dynamic and plyometric lunges – straight forward and diagonal, butterfly, sprinter arms, explosive steps, step jumps, diagonal sprint steps.

*Shooting and stability*: Torso rotations, crunches, bench press without weights, shot pose, upswing pose, various types of shots and slap shots in static, dynamic and explosive motion, as well as different types of planking exercises.

*Jumping ability*: Squats with different ranges of motion, squats with pulses, straight jumps, squat jumps, jumps over box, CMJs.

*Isokinetic force*: Static and dynamic squats, explosive squat pushes, high skips – squat – jump squat combinations, slow and fast squats, tap the floor, alternating lunge jumps.

Electrode-straps were attached to upper arms, upper legs and buttocks and a vest was done to stimulate back and abdominal muscles. Pulse currents of 85 Hz frequency with a pulse width of 350 μs were used. A duty cycle of 50% with a contraction time of 4 s was applied. At the beginning of each session the individual daily maximum tolerated pain intensity was adjusted and a consecutive increase throughout the training period was sought. The minimum level was set to be at least 75% of last session’s intensity score.

### Testing

Shot speed, jumping ability, 10-m sprint time, maximum isokinetic force at 60 and 300°/s and the subjective performance according to a questionnaire was recorded. The measurements were taken at baseline, after 6 and after 12 weeks. The results after the WB-EMS pause phase were tested 16 weeks after the start of the training as a follow up measure. All data were collected in accordance to the training- and match time table of the subjects. Additionally, the appointments were scheduled in adequate distance to previous WB-EMS sessions to achieve equivalent physiological initial conditions for all players.

According to the cross-over strategy chosen for this trial, there were two study periods of equal length, divided by a wash-out phase. Group A had WB-EMS in addition to regular ice hockey training during period 1 and regular training only in period 2, whereas the schedule for Group B was arranged in reverse.

**FIGURE 1:**
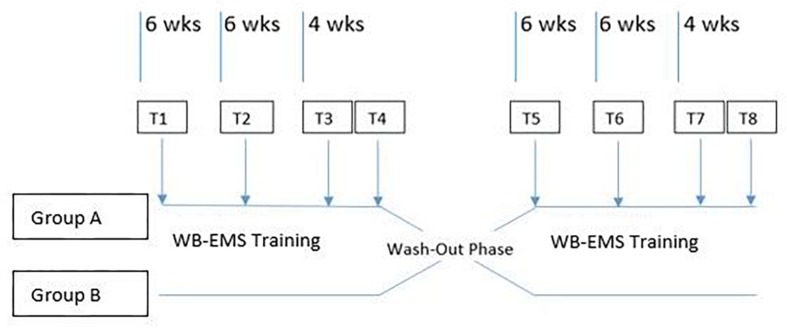
Scheme of cross-over study course with consecutive wash-out phase between period 1 and period 2.

Period 1 started with the initial physical examination and testing on T1 corresponding baseline-results. After a 6 week WB-EMS training phase in Group A and exclusive ice hockey training in Group B, the second data collection followed at T2. Re-measurement of the variables took place after 12 weeks. The final measurement T4 after 16 and 4 weeks of WB-EMS pause respectively, was a follow-up measurement to evaluate the retention of training effects. After a wash-out phase of 3 months, period 2 ensued analog with Group B undergoing training with EMS (Figure 1).

T1 and T3, such as T5 and T7 were used as reference measurements. T2 and T6 were used to illustrate the value development. T4 and T8 were collected to assess the degree of retention of any training effects. Data of all participants of both groups were collected at each measurement.

Since fewer athletes participated in measurement T6 than in the other measurements, this measurement is not considered representative.

### Shot Speed

The shot speed in km/h was measured by means of an unmodulated continuous wave sport radar “Ballspeedometer 1503.” This sensor was centrally located just behind the net of the goal and all shots were targeted at the sensor. For the test, subjects performed three slap shots from a distance of 10 m and the average shot speed was calculated from these three individual shots.

### Jumping Ability

Individual maximum jumping power and maximum jump height in the CMJ were measured using the Leonardo Mechanograph GRFP (Novotec Medical GmbH, Pforzheim, Germany) with a sampling rate of 800 Hz. Ground reaction forces were recorded using a measuring pressure plate via the springboard and then evaluated using the accompanying LabVIEW software. For testing, the players stood with both legs in the predetermined areas of the plate and performed three valid consecutive CMJs. Sufficient time was provided between each CMJ. In terms of implementation, we decided to include arm swinging.

### Sprint

The maximum sprint time in seconds for a 10 m track was recorded using Model RL S1c photocell (ALGE-Timing GmbH, Lustenau, Germany). The second sensor-reflector pair of the photocell was mounted centrally above the blue line. From there, the 10 m sprint distance was measured toward the short side of the boards, where then the first sensor-reflector pair was placed. The run-up took place from the red baseline. All participants lined up in turn. After this predetermined start-up distance each player passed the photocell construction of a distance of 10 m a total of three times. Sufficient recovery time was provided in between the rounds. Sprint times were determined at hip level.

### Isokinetic Test

The maximum isokinetic force at 60 and 300°/s measurement of the knee extensors in Nm was determined by IsoMed 2000 (D. & R. Ferstl GmbH, Hemau, Germany). After a 5–10 min warm-up on the bicycle or treadmill ergometer, which was set as standardized choice for each player, a test round with approximately 75% of subjective sub maximum power took place at each angular velocity, followed by the actual test run. Five repetitions were required for the maximum force test at 300°/s and four passes were required for the subsequent maximum force measurement at 60°/s, as set by default of IsoMed 2000. Range of motion for the knee joint was set to 90° with start position at 90° and end position at 0. The best performance out of all passes was counted. Between each pass the athletes were given 45 s to rest. The subjects were equipped with straps on hip, chest, leg, and thighs, to minimize influencing movements.

### Subjective Questionnaire

The survey was conducted before each WB-EMS series and at the end of both periods. Each participant completed the questionnaire including weight development, limb circumferences, injuries and self-assessment of sprint ability, shoot speed, jump performance, leg strength, body shape, fitness level, mood, safety on ice, stability, and overall satisfaction.

### Statistical Analysis

Estimates and caseload calculations were made within statistical planning based on the work of other research groups and issues, as there had been no experience and data on the variability of values and possible intervention effects.

To reach a power of at least 90%, a caseload of a minimum of 25 participants was needed. All values were calculated using mean value and standard deviation. Each variable that underwent three passes during testing was standardized as an average value. Unrealistic values >2 SD or errors were removed from the calculation. The comparisons for verified differences were used as unilateral paired *t*-tests for unconnected samples. A normal distribution of the values was assumed. For data evaluation and interpretation, a power of 90% was estimated with significance accepted for *p*-values ≤0.05. The statistical support was provided by a statistician of the Institute for Medical Information Processing, Biometry and Epidemiology of the LMU Munich. All statistical methods were performed with the SPSS software (IBM Corp., Armonk, NY, United States).

## Results

### Vertical Jump Power

**FIGURE 2:**
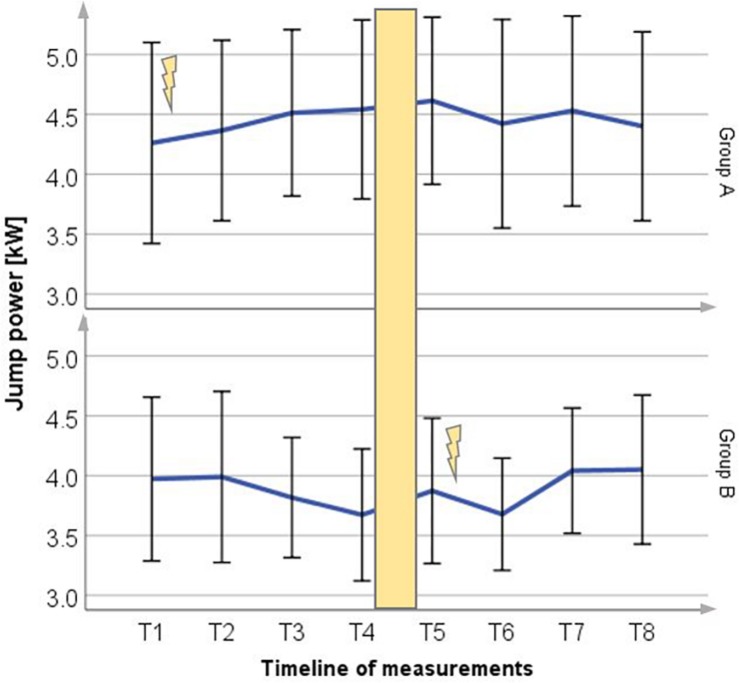
Mean (±SD) change in jump power for Group A period 1 and Group B period 2, compared to each control group (*p* = 0.04). Lightning bolt represents each respective WB-EMS period; yellow area = wash-out phase.

Results for vertical jump power, obtained during 16 weeks of testing, are shown in Figure 2 for both groups. In all following figures MV denotes mean value, the lightning bolt illustrates the respective intervention period. After WB-EMS training jump power increased significantly in Group A to 5.9% (251.5 ± 226.6 W) and 4.4% (168.5 ± 314.9 W) in Group B, respectively (*p* < 0.05). In corresponding phases of exclusive ice hockey training, jump power values decreased in both groups.

### Vertical Jump Height

**FIGURE 3:**
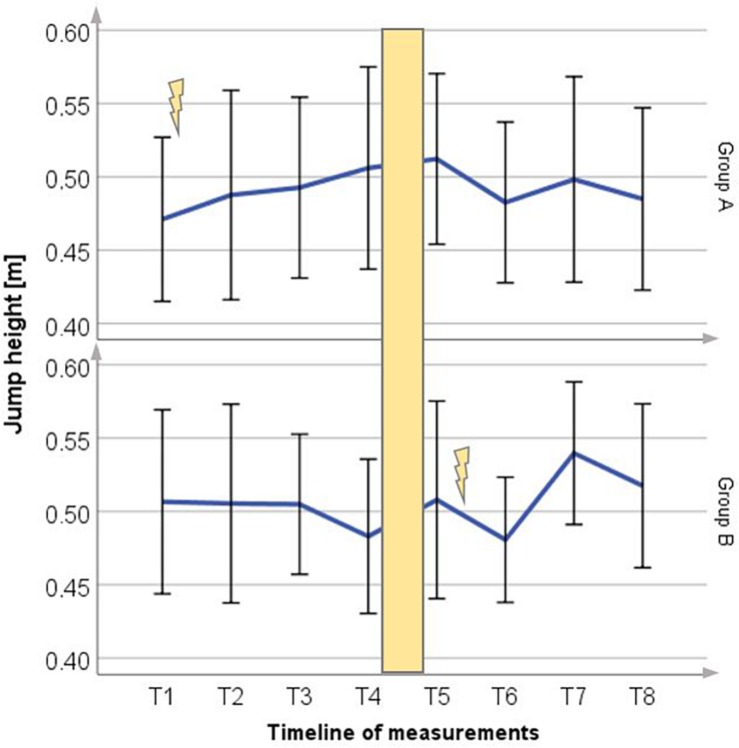
Mean (±SD) change in jump height for Group A period 1 and Group B period 2. Lightning bolt represents each respective WB-EMS period; yellow area = wash-out phase.

Results for vertical jump height obtained during the 16 weeks testing period are shown in Figure 3 for both groups. Jump height increased in Group A to 4.6% (2.16 ± 3.34 cm) and 6.3% (3.18 ± 4.54 cm) in Group B, respectively. In corresponding phases of exclusive ice hockey training jump height values decreased in both groups.

### Sprint Time

**FIGURE 4:**
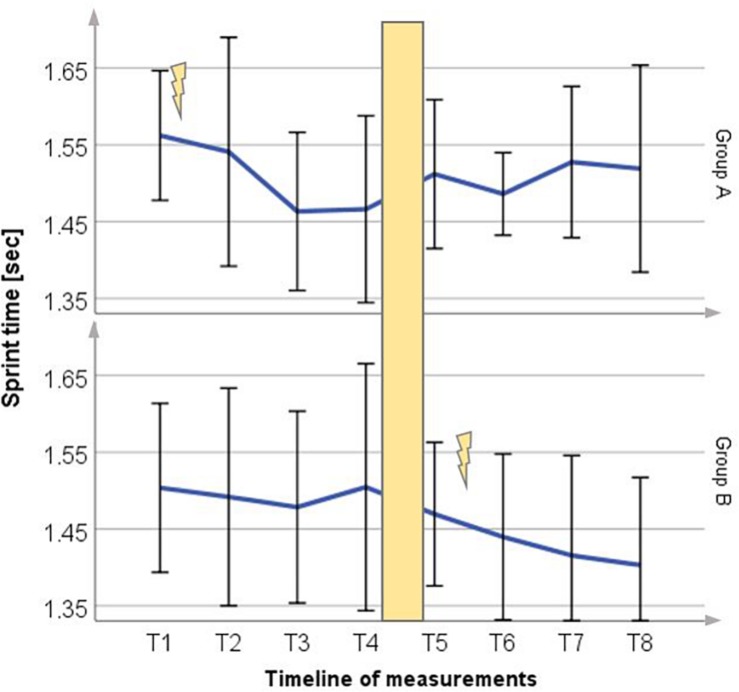
Mean (±SD) change in sprint time for Group A period 1 and Group B period 2, compared to each control group (*p* = 0.02). Lightning bolt represents each respective WB-EMS period; yellow area = wash-out phase.

The 10 m skating time decreased significantly for Group A to 6.3% (0.1 ± 0.06 s) and Group B to 3.7% (0.05 ± 0.06 s) shown in Figure 4. Without WB-EMS training the sprint time results showed an increase in Group A and no significant changes in Group B.

### Maximum Force at 60°/s

**FIGURE 5:**
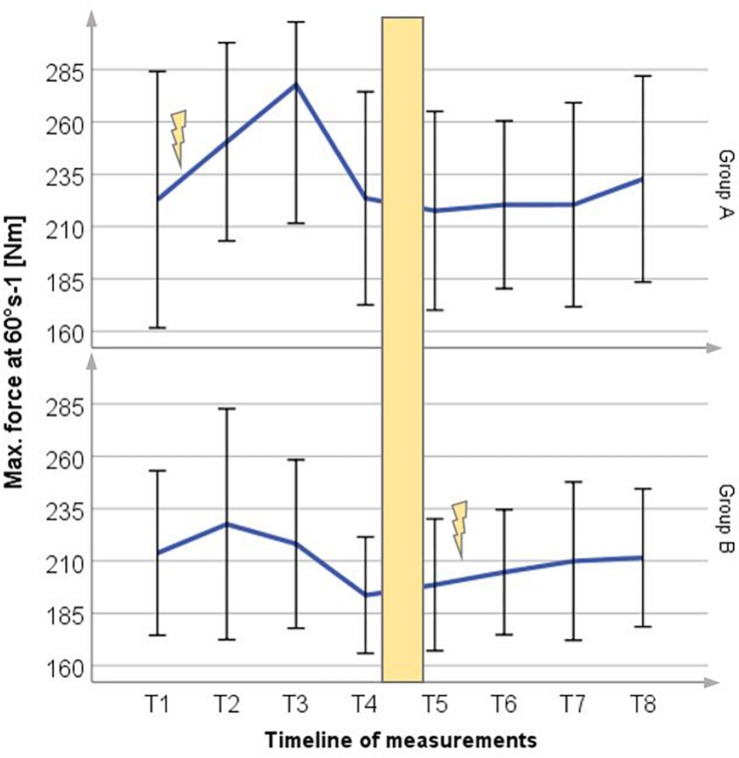
Mean (±SD) change in maximum voluntary force at 60°/s for Group A period 1 and Group B period 2. Lightning bolt represents each respective WB-EMS period; yellow area = wash-out phase.

After 12 weeks of WB-EMS training the isokinetic torque at 60°/s increased to 24.6% (54.86 ± 69.07 Nm) for Group A and to 5.7% (11.33 ± 21.88 Nm) for Group B as shown in Figure 5. Without WB-EMS training the results of each group showed no significant changes.

### Maximum Force at 300°/s

**FIGURE 6:**
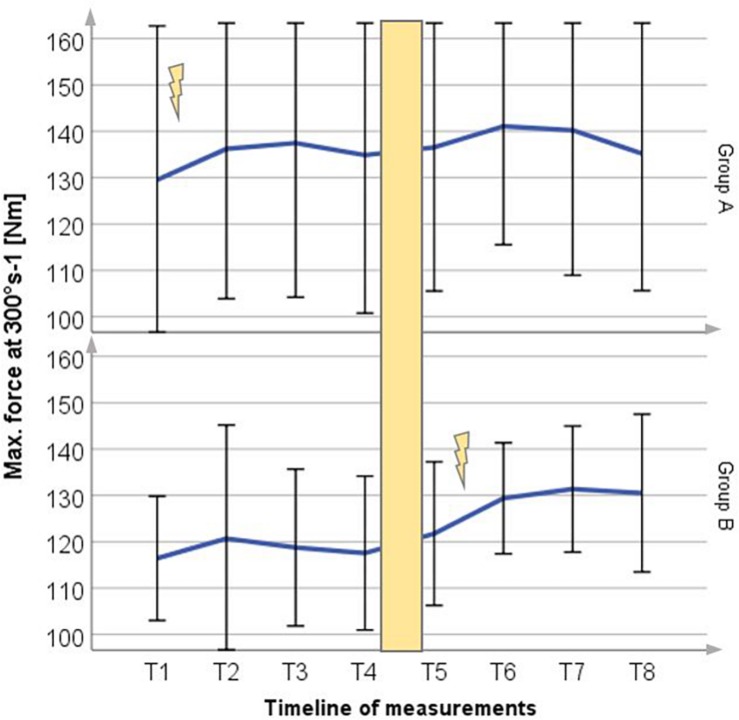
Mean (±SD) change in maximum voluntary force at 300°/s for Group A period 1 and Group B period 2, compared to each control group (*p* = 0.02). Lightning bolt represents each respective WB-EMS period; yellow area = wash-out phase.

After 12 weeks of EMS training, the isokinetic torque at 300°/s increased significantly (*p* < 0.05) (Figure 6) for Group A to 6.1% (7.94 ± 20.73 Nm) and Group B to 7.9% (9.61 ± 13.57 Nm). When comparing torque changes after the 12-week period, WB-EMS groups had significantly higher torque increases than the control groups. Without WB-EMS training the results of each group showed no significant changes.

### Shot Speed

After 12 weeks of WB-EMS training no changes were detected for both groups (±0; *p* < 0.05).

### Subjective Questionnaire

The evaluation showed an increased self-assessment of all points after WB-EMS-training, except for the mood, which remained approximately constant in group A during the intervention. Regarding the injury rate, no new hockey-related injuries were detected during the study periods, although four injuries due to hobby or work were registered.

### Comparison of Leagues

Comparing the two leagues, every parameter improved after WB-EMS training, except shot speed and maximum force at 300°/s considering hobby league players of Group A. Looking at the averaged scores, hobbyists achieved higher results when it came to jump power (6.35 vs. 3.25%), sprint time (7.4 vs. 4.6%), maximum force at 300°/s (7 vs. 5.85%), and jump height (11 vs. 2.3%) albeit hobby players represented a comparatively smaller proportion of our study population. District league players showed higher values for max force at 60°/s after WB-EMS training (16.1 vs. 11.75%).

## Discussion

In our study, a 12-week WB-EMS training in addition to normal ice hockey training of leisure players showed a comparable development of the sprint time (Filipovic et al., 2016), (e.g., by 4.8% in elite ice hockey players) (Brocherie et al., 2005), but less improvement in maximum force at 60 and 300°/s of the quadriceps (Maffiuletti et al., 2000; Brocherie et al., 2005; Billot et al., 2010; Filipovic et al., 2016), (e.g., 41.3 ± 37.6% at 60°/s and 49.2 ± 48.9% at 300°/s in elite ice hockey players) (Brocherie et al., 2005) and a contrary trend in terms of jump height (Brocherie et al., 2005). The different findings of our study in comparison to other authors regarding leg strength could be explained by our differing training concept. In contrast to other studies we used whole-body EMS training and did not only address the M. quadriceps physically. Therefore, overall less time was spent on stimulating the leg extensor. Furthermore, our training plan involved mainly static exercises for the first 6 weeks, followed by dynamic exercises only in the second 6 weeks, which could also be a cause for the lower improvement of the maximum force at 300°/s. All in all, the trend of the value development of sprint time, maximum force at 60 and 300°/s is consistent to the results of previous studies.

In addition to the variables described, we also tested jump power, which increased by 5.15% and shot speed. Regarding the evaluation of shot velocity no changes were observed in our study. At the present time, there are no studies evaluating the effect of EMS training on shot velocity. The slap shot is a very complex motion and highly depends on the level of technique and experience of the player. The lack of improvement could be partly explained by factors such as technique or motivation (Babault et al., 2007). Also, despite sport specific training exercises, improvements in complex motions are restricted (Micke et al., 2018).

Regarding the jump performance, many findings show no change or a decrease of jump performance after EMS training. At best little improvement was found after the end of the stimulation period. Ten days after the completion of the stimulation sessions and continued volleyball training jump performance improved by 5–6% (Malatesta et al., 2003). In line with this, elite basketball players did not experience any significant changes in CMJ height after local EMS training. A subsequent consecutive 4-week EMS break with basketball practice only also led to a significant gain in jump performance of 17% (Maffiuletti et al., 2000). Local EMS training on soccer players presented no significant changes in CMJ (Billot et al., 2010). In professional ice hockey players, local EMS training on the quadriceps resulted in a 6.1% decrease of vertical jump height (2.1 ± 2.0 cm) (Brocherie et al., 2005). Those results are contradictory to the findings of other researchers who found improvements after the end of an EMS period (Filipovic et al., 2016). For instance, a WB-EMS study with runners showed improved jumping skills (0.02 ± 0.02 m) after WB-EMS training, compared to a control group (Amaro-Gahete et al., 2018b), which is in accordance with our findings. Ameliorations in complex movements like jumping are limited (Micke et al., 2018). Authors state that performance in complex movements requires some time of specific training before the positive effects of local EMS can be observed (Maffiuletti et al., 2000). Therefore, longer EMS training sessions could be more efficient on jumping skills. Most authors used shorter EMS training periods. Our study involved 12 weeks of WB-EMS training. This could be one explanation why jumping performance increased at the end of our study. Micke et al. (2018) postulated that absence of improvement under WB-EMS training could be due to training without sport specific movements. It has been shown that specific exercises lead to higher gains in jump performance of runners compared to traditional WB-EMS training (Amaro-Gahete et al., 2018a). Therefore, we decided to use several specific exercises for ice hockey players.

Research has shown that there is a connection between weight training and a reduction in injury rate. Targeted strength training is useful as injury prevention (Tyler et al., 2002; Weisskopf, 2010). Moreover, studies show that training with local EMS and strength training have similar effects in terms of strength gains (Simpson and Willoughby, 1988; Colson et al., 2009). Our study questionnaire gave evidence to higher self-assessments in terms of safety and stability on the ice. Strength gains and increase of individual subjective performance could give an indirect effect on injury rate. Accordingly, in a large population, an effect on the injury rate could be expected.

Currently, the data on WB-EMS in leisure sports is limited. In our study, significant training effects were recorded after WB-EMS training. Regarding the influence of competitive status, our conclusion is that, by taking in account the above arguments, there is an even greater potential for improvement for hobby sportsmen. Because of the lower training potential of professional athletes, their training effect should be less pronounced than with leisure athletes.

To summarize, we found additional WB-EMS training suitable as a supplement to normal training for lower league ice hockey athletes. It improved their physical performance in strength and speed parameters. In a larger study population, it might possibly serve as injury prophylaxis.

## Data Availability

All datasets generated for this study are included in the manuscript and/or the supplementary files.

## Ethics Statement

The study was approved by the Ethics Committee of the Ludwig Maximilian University of Munich. It was registered with the German clinical trials register (ID: DRKS00012249) and is therefore listed in the International Clinical Trials Registry of the World Health Organization.

## Author Contributions

ES trained the athletes, collected the data, analyzed the data, and prepared the manuscript. CB, HS-G, and VJ helped to prepare, translate, and review the manuscript. AP performed the isokinetic measurements. BW planned and supervised the study, prepared and wrote the manuscript.

## Conflict of Interest Statement

The authors declare that the research was conducted in the absence of any commercial or financial relationships that could be construed as a potential conflict of interest.
